# Role of *interleukin 1β* and *interleukin 10* variants on ocular toxoplasmosis in Brazilian individuals

**DOI:** 10.3389/fopht.2023.1183167

**Published:** 2023-06-29

**Authors:** Warlen Miiller Rocha Araujo, Christiane Maria Ayo, Mariana Previato, Geraldo Magela de Faria, Fábio Batista Frederico, Rubens Camargo Siqueira, Gildásio Castello de Almeida, Vera Lúcia Pereira-Chioccola, Luiz Carlos de Mattos, Cinara Cássia Brandão

**Affiliations:** ^1^ FAMERP Toxoplasma Research Group, Department of Molecular Biology, Faculdade de Medicina de São José do Rio Preto (FAMERP), São Paulo, Brazil; ^2^ Ophthalmology Outpatient Clinic, Fundação Faculdade Regional de Medicina, Hospital de Base da Fundação Faculdade Regional de Medicina (HB-FUNFARME), São Paulo, Brazil; ^3^ Department of Parasitology, Instituto Adolfo Lutz—São Paulo, São Paulo, Brazil

**Keywords:** ocular toxoplasmosis, cytokines, interleukins, genetic polymorphisms, IL1β and IL10

## Abstract

**Introduction:**

Ocular toxoplasmosis (OT) is an intraocular inflammation caused by *Toxoplasma gondii* infection that affects the retina and choroid, giving rise to posterior uveitis. Genetic polymorphisms in cytokine genes may exert influence in the expression of these molecules and play a significant role in inflammatory responses and susceptibility to OT. The aim of this study was to evaluate the role of polymorphisms rs16944 (–511 C > T) of the *interleukin (IL) 1β* gene and rs1800896 (–1082 G > A) of the *IL10* gene on OT in Brazilian individuals with a serologic diagnosis of *T. gondii* and after conducting fundoscopic exams.

**Methods:**

Participants with a positive serology were classified into two distinct groups according to the presence (G1; *n* = 110) or absence (G2; *n* = 104) of OT. The control group (G3) consisted of individuals without the infection (*n *= 108).

**Results:**

It was observed that the C/C genotype of the *IL1β* gene polymorphism was a protective factor for OT (*p* = 0.02, OR = 0.28, 95% CI 0.08–0.78 for G1 vs. G2; *p* = 0.03; OR = 0.29, 95% CI 0.09–0.82 for G1 vs. G3), according to the recessive inheritance model.

**Conclusions:**

The -511C.T polymorphisms of the *IL1β* gene seems to play an important role in the pathogenesis of OT in Brazilian individuals.

## Introduction

Ocular toxoplasmosis (OT) is an intraocular inflammation caused by *Toxoplasma gondii* infection that affects the retina and choroid (retinochoroiditis), giving rise to posterior uveitis. It can be of congenital origin or (in most cases) acquired after birth, and old lesions can reactivate at any time (1–4). In some cases, OT can leave serious sequelae, including complete loss of vision when the optic nerve is affected (1). Studies have shown that the prevalence and severity of OT are much higher in Brazil than in many other parts of the world, such as Europe and North America (5, 6).

In addition to the infective strain of the parasite, host genetic factors related to immune response can determine the final clinical outcome of infection (7, 8). Cytokines are crucial to the development and functioning of immune responses because they are important signaling molecules involved in cellular communication. Some of these molecules may play an important role in immune protection, but can also be pathological when unregulated (9).

Regarding toxoplasmosis, specific cytokines are important in the pathogenic process of OT (10). Genotypes and promoter sequences of genes related to the coding of cytokines have been associated with the development of human OT (11–17), as genetic polymorphisms in cytokine genes may exert influence on the expression of these molecules (14, 18) and play a significant role in inflammatory responses and susceptibility to OT.

IL-1β and IL-10 are cytokines that participate in the host immune response. *T. gondii*-infected human retinal pigment epithelium cells produce cytokines that activate neutrophils and lead to the production of proinflammatory cytokines such as IL-1β (19). Although inflammatory responses are critical for host defense against the parasite, excessive inflammation can result in tissue damage associated with OT. Furthermore, *T. gondii* can induce IL-1β production by monocytes depending on the specific strain (20). In contrast, an important role for the anti-inflammatory cytokine IL-10 in modulating ocular disease has been demonstrated, as this cytokine contributes to the balance between protective and pathological T-cell responses. (21). The *IL1β* gene is located on chromosome 2. The –511 polymorphism, located in the *IL1β* promoter region, affects the regulatory sites of *IL1-β*, thus influencing gene expression and circulatory levels of this cytokine. The literature shows that the T allele was associated with lower levels of mRNA expression for *IL1-β* (22). The *IL10* gene is located on chromosome 1. The A allele of the –1082 polymorphism in the *IL10* promoter region was associated with lower levels of production of IL-10 (23).

Considering that the expression of cytokines is regulated by genetic variants and evidence suggests that cytokine gene polymorphisms may contribute to the presence of ocular lesions in patients infected by *T. gondii*, the aim of this study was to evaluate the role of polymorphisms rs16944 (–511 C > T) of the *IL1β* gene and rs1800896 (–1082 G > A) of the *IL10* gene in OT in Brazilian individuals.

## Materials and methods

### Ethics information

This study was approved by the Research Ethics Committee of the Medicine School in São José do Rio Preto (CAAE 32259714.8.00005415). All individuals who agreed to participate signed informed consent forms.

### Composition of the study

This was a case–control study. Between January 2015 and December 2017, a total of 322 consecutive and unrelated individuals seen at the Retinopathy Outpatient Clinic of the Specialty Hospital, Faculty of Medicine de São José do Rio Preto (HB-FUNFARME), participated in this study. These patients were classified into three groups. Group 1 (G1) contained 110 individuals with a positive serology for *T. gondii* and with OT present. Group 2 (G2) contained 104 individuals with a positive serology for *T. gondii* and the ocular diseases other than toxoplasmosis present, such as age-related macular degeneration (24.0%), cataracts (19.2%), retinal detachment (14.4%), diabetic retinopathy (10.6%), glaucoma (6.7%), macular edema (3.8%), pterygium (3.8%), optic neuropathy (2.9%), macular atrophy (1.9%), and other ocular diseases (12.5%). Group 3 (G3) contained 108 individuals with a non-reactive serology for *T. gondii* (control group) who attended the clinic to check their visual acuity.

All individuals who participated in this study were residents of municipalities in the northwest region of the State of São Paulo—located in the southeast region of Brazil, between 20°49′13″S and 49°22′47″W. The individuals were monitored and evaluated for clinical symptoms, *T. gondii* serology, and epidemiological data. In addition, they were defined as belonging to a population of mixed ethnicity owing to the high heterogeneity of the Brazilian population (24).

The inclusion criteria for individuals with OT were a positive laboratory diagnosis for *T. gondii* and the presence of scars/eye lesions due to toxoplasmosis. Inclusion criteria for individuals without OT were a positive laboratory diagnosis for *T. gondii* and an absence of eye scars/lesions due to toxoplasmosis. The inclusion criterion for the individuals who made up the control group was a negative laboratory diagnosis for *T. gondii*.

Exclusion criteria were individuals with other infectious and parasitic diseases, individuals with some type of mental disability, individuals with blood dyscrasia and who used oral anticoagulants, and individuals related to patients already enrolled.

### Serologic diagnosis

Blood samples were collected in tubes without anticoagulant to obtain serum. The presence of anti-*T. gondii* IgM and IgG was confirmed by enzyme-linked immunosorbent assay (ELISA) (DiaSorin S.p.A., Saluggia, Italy) using the ETI-TOXOK-M reverse plus kit for IgM and the ETI-TOXOK-G plus kit for IgG, and by enzyme-linked fluorescence assay (ELFA) (BioMérieux, Marcy-l’Étoile, France) using the Vidas® Toxo IgM (TXM) kit for screening for IgM; and the Vidas® Toxo IgG II (TXG) kit for screening for IgG. All tests were performed according to the manufacturer’s instructions.

### Clinical diagnosis

All individuals were clinically evaluated by two experienced ophthalmologists using retinography according to the Early Treatment Diabetic Retinopathy Study Report (ETDRS) standards and criteria (25, 26). As no invasive test was performed, this was a presumptive diagnosis for OT.

### *IL1β* and *IL10* genotyping

Genomic DNA was extracted using a silica column QIAamp Blood Mini kit (Qiagen) from peripheral blood collected with ethylenediaminetetraacetic acid (EDTA). DNA quantity and quality were measured using a spectrophotometer (BioTek, Epoch Instruments).

The respective cytokines’ polymorphisms were selected based on data from previous studies carried out in the context of ocular toxoplasmosis or other human diseases. In addition, single nucleotide polymorphisms (SNPs) were chosen based on their predicted effect on the level of gene expression, which was obtained through STIFF tool analysis, available at the Ensembl genome browser (https://www.ensembl.org/index.html), and through previous reports of an association with any disease condition. Genotyping for the identification of the rs16944 (–511 C/T) polymorphism of the *IL1β* gene and rs1800896 (–1082 G/A) polymorphism of the *IL10* gene was performed using the PCR-restriction fragment length polymorphism (PCR-RFLP) technique. The primer pair used for the *IL1β* gene was sense 5*′*-TGGCATTGATCTGGTTCATC-3′ and antisense 5′-GTTTAGGAATCTTCCCACTT-3′, and the primer pair used for the *IL10* gene was sense 5′-CCAAGACAACACTACTAAGGCTCCTTT-3′ and antisense 5′-GCTTCTTATATGCTAGTCAGGTA-3′. The PCR reaction conditions were previously described by Piatto et al. (27). The PCR products were digested with *Eco*88I (Thermo Fisher Scientific, Waltham, MA, USA) for the *IL1β* gene and with the *Faq*I (Thermo Fisher Scientific) for the *IL10* gene. The digested PCR products were subsequently separated by agarose gel electrophoresis and detected using the SYBR™ Green nucleic acid gel stain (Invitrogen Life Technologies, Grand Island, NY, USA).

### Statistical analysis

Allele and genotypic frequencies were obtained by direct counting, and the Hardy–Weinberg equilibrium (HWE) was verified according to the method described by Guo and Thompson (28). Comparisons of these frequencies between the groups of patients were performed using the chi-squared test (χ^2^) with Yates’ correction or by Fisher’s exact test, using a 3 × 3 contingency table for genotypes and a 3 × 2 contingency table for alleles. The genotypic frequencies were evaluated against each other using the models of dominant, recessive, and codominant inheritance, by means of a 2 × 2 contingency table. The odds ratio (OR) was calculated to verify association estimates using the GraphPad InStat program (InStat 3.06, 2003). The mean ages were compared using a *t*-test. All tests produced a 95% confidence interval (CI) and differences with *p*-values < 0.05 were considered statistically significant.

## Results

The general characteristics of the study individuals are shown in **Table 1**. Of the total number of individuals analyzed in G1, 60 (54.5%) were male and 50 (45.5%) were female; in G2, 50 (48.0%) were male and 54 (52.0%) were female; and in G3, 55 (51.9%) were male and 53 (49.1%) were female. The mean age was statistically different between the groups (G1 vs. G2 vs. G3: *p* < 0.0001; G1 vs. G2: *p* < 0.0001, *t* = 5.38; G1 vs. G3: *p* = 0.002, *t* = 3.04; G2 vs. G3: *p* < 0.0001, *t* = 10.25); in G1 the mean age was 72.7 years (± 21.4 years), in G2 it was 56.9 years (± 17.0 years), and in G3 it was 35.2 years (± 13.6 years).

**Table 1:** General characteristics of individuals infected by *Toxoplasma gondii* with and without ocular toxoplasmosis and of individuals without the infection.

Characteristic	G1 (*n* = 110)	G2 (*n* = 104)	G3 (*n* = 108)	*p*-value
Sex, *n* (%)				
**Male**	60 (54.5)	50 (48.1)	55 (51.0)	NS
**Female**	50 (45.5)	54 (51.9)	53 (49.0)	
Age (years)				
**Mean ± SD**	72.7 ± 21.4	56.9 ± 17	35.2 ± 13.6	< 0.0001^a^
**Range**	10–95	19–88	18–80	
**Median**	37	58.5	33	

G1: individuals with OT; G2: individuals without OT; G3: control group. NS: non-significant. a G1 versus G2 versus G3.

p < 0.0001; t = 5.38 (G1 versus G2).

p = 0.002; t = 3.04 (G1 versus G3).

p < 0.0001; t = 10.25 (G2 versus G3)

The distribution of alleles and genotypes for the –511 *IL1β* and –1082 *IL10* polymorphisms was in the HWE in the control group (*p* > 0.05).

In relation to the *IL1β* gene polymorphism (position –511), there was a statistically significant difference between the groups in genotype frequency (*p* = 0.0028; χ^2^ = 16.13; gl = 4). According to the inheritance models, a significant difference was found using the recessive model for the C allele as a protective factor against OT, showing that it would be necessary to have two C alleles (C/C genotype) to exert protection against the occurrence of OT (*p* = 0.02, OR = 0.28, 95% CI 0.08–0.78 for G1 vs. G2; and *p* = 0.03, OR = 0.29, 95% CI 0.09–0.82 for G1 vs. G3). The alleles expressed as heterozygous (T/C genotype) were more frequent in G1 than in G2 (*p* = 0.056, OR = 2.05, 95% CI 1.03–4.16) and G3 (*p* = 0.0004, OR = 3.30, 95% CI 1.72–6.52). The presence of two T alleles (T/T genotype) was more frequent in G3 than in G1 (*p* = 0.02, OR = 0.39, 95% CI 0.17–0.83) and G2 (*p* = 0.059, OR = 0.45, 95% CI 0.20–0.96). However, both the T/C and T/T genotype were not associated with the occurrence of OT, as shown by the codominant and dominant inheritance models, respectively (**Table 2**).

**Table 2:** Genotype and allele distribution for *IL1β* –511 and IL10 –1082 polymorphisms in individuals infected by *Toxoplasma gondii* with and without ocular toxoplasmosis and in individuals without the infection.

Genotypes/alleles	G1, *n* (%)	G2, *n* (%)	G3, *n* (%)	*p*-value	OR (95% CI)
	(*N* = 110)	(*N* = 104)	(*N* = 108)		
IL1β rs16944 (–511 C > T)					
C/C	5 (4.5)	15 (14.5)	15 (13.9)	0.0028^a^ χ^2^ = 16.13	
C/T	94 (85.5)	77 (74.0)	69 (63.9)		
T/T	11 (10.0)	12 (11.5)	24 (22.2)		
C/C	5 (4.5)	15 (14.5)	15 (13.9)	0.02^b^	0.28 (0.08–0.78)
T/T + C/T	105 (95.5)	89 (85.5)	93 (86.1)	0.03^c^	0.29 (0.09–0.82)
T/T	11 (10.0)	12 (11.5)	24 (22.2)	0.02^c^ 0.059^d^	0.39 (0.17–0.83) 0.45 (0.20–0.96)
C/C + C/T	99 (90.0)	92 (88.5)	84 (77.8)		
C/T	94 (85.5)	77 (74.0)	69 (63.9)	0.056^b^	2.05 (1.03–4.16)
T/T + C/C	16 (14.5)	27 (26.0)	39 (36.1)	0.0004^c^	3.30 (1.72–6.52)
C	104 (47.3)	107 (51.4)	99 (45.9)	NS^a^	
T	116 (52.7)	101 (48.6)	117 (54.1)		
IL10 rs1800896 (–1082 G > A)					
G/G	4 (3.6)	6 (5.7)	1 (0.9)	NS^a^	
G/A	104 (94.6)	94 (90.3)	106 (98.2)		
A/A	2 (1.8)	4 (4.0)	1 (0.9)		
A/A	2 (1.8)	4 (4.0)	1 (0.9)	NS^b,c,d^	
G/G + G/A	108 (98.2)	100 (96.0)	107 (99.1)		
G/G	4 (3.6)	6 (5.7)	1 (0.9)	NS^b,c,d^	
A/A + G/A	106 (96.4)	98 (94.3)	107 (99.1)		
G/A	104 (94.6)	94 (90.3)	106 (98.2)	0.03^d^	0.17 (0.02–0.75)
G/G + A/A	6 (5.4)	10 (9.7)	2 (1.8)		
G	112 (50.9)	106 (50.9)	108 (50.0)	NS^a^	
A	108 (49.1)	102 (49.1)	108 (50.0)		

CI, confidence interval; individuals with OT; G2, individuals without OT; G3, control group; NS, non-significant; OR, odds ratio; OT, ocular toxoplasmosis.

a G1 vs. G2 vs. G3;

b G1 vs. G2;

c G1 vs. G3;

d G2 vs. G3.

The frequencies of genotypes and alleles were compared using the chi-squared test for 3 × 3 and 3 × 2 contingency tables, respectively. Frequencies of genotypes according to models of inheritance were compared using the chi-squared test for 2 × 2 contingency tables.

The comparison of G1 + G2 with G3 showed that the wild-type homozygote (T/T) genotype of the *IL1β* –511 polymorphism was more frequent in G3 (*p* = 0.007, OR = 0.042, 95% CI 0.22–0.78) and the heterozygous genotype was more frequent in individuals with the infection (G1 + G2) (*p* = 0.002, OR = 2.24, 95% CI 1.33–3.76) (**Supplementary Table 1**).

Regarding the –1082 polymorphism of the *IL10* gene, statistically significant differences were not found for genotype and allele distribution between the groups. Considering the analyses through the inheritance models, the codominant model showed that the heterozygous G/A genotype was more frequent in G3 than in G2 (*p* = 0.03, OR = 0.17, 95% CI 0.02–0.75) (**Table 2**). This same observation was found in the comparison of G1 + G2 with G3, in which the heterozygous genotype was more frequent in patients without the infection (G3) (*p* = 0.04, OR = 0.23, 95% CI 0.05–1.03) (**Supplementary Table 1**).

## Discussion

Despite numerous discoveries in recent years, OT remains a poorly understood disease, despite its epidemiological relevance (29). The importance of age in the clinical course of OT has been reported, and studies have shown that the disease most often affects patients between the second and fourth decades of life (2, 30, 31). However, OT can develop at any time in life (2, 32) and older patients appear to be at greater risk of developing eye injuries (2, 3, 5).

In this study, the group of individuals with eye lesions resulting from a *T. gondii* infection (G1) had a higher mean age than the other groups (G2 and G3). Ferreira et al. (33) demonstrated that a higher average age is a risk factor for the development of OT (33). In addition, as most cases of OT occur due to infections acquired after birth (1), the difference observed in terms of age between the groups in this study can be attributed to longer exposure to the parasite, both for the development of OT and for the acquisition of infection. A possible explanation for the higher mean age observed in G2 than in G3 is that eye diseases, other than OT, are more prevalent in older patients (34).

CD4+ and CD8+ T cells, B cells, and macrophages have been identified as the effector cells that attack and destroy infected neurons in the retina. The expansion, migration, and activation of these cells may be associated with cytokine signaling functions (29). Individuals with OT present abnormal expression in the levels of some cytokines (i.e., elevated or reduced levels), and these changes in expression levels may be associated with polymorphisms in cytokine genes (14, 18).

The data obtained in this study showed that the homozygous C/C genotype of the *IL1β* gene rs16944 polymorphism was a protective factor for OT in a Brazilian population; this finding has never been previously published. According to the literature, the C allele of this mutation is related to the high expression of the gene, i.e., increased production of the cytokine (11, 15–17, 35–37). Therefore, we suggest that the homozygous presence of the C allele may generate an efficient immune response for parasite control, so that individuals with this polymorphism are protected against the occurrence of OT.

IL-1β upregulates other cytokines that are known to be potent neutrophil chemoattractants (19). Therefore, neutrophil recruitment to the *T. gondii*-infected human retinal pigment epithelium cells, enhanced by IL-1β, may play an important role in the pathogenesis of the infection. It is known that IL-1β can modulate pro-inflammatory T helper (Th) 1 (Th1) response by impeding IL-6-induced phosphorylation of signal transducer and activator of transcription 1 (38). A regulated Th1 response is necessary for the efficient control of the parasites and to avoid tissue damage. High levels of IL-1β inhibit the growth of the parasite in endothelial cells in the retinal region (10, 36). Machado et al. (39) showed that in newborns with a positive serology for *T. gondii*, the increase in IL-1β production was an important indicator of protection against or resolution of eye injury. In addition, Brunton et al. (36) demonstrated that low levels of IL-1β expression can result in OT. However, a recent study conducted by Naranjo-Galvis et al. in Colombian subjects found no association between the rs16944 polymorphism in the –511 region of the *IL1β* gene and OT (17).

In this study, the genotypes and alleles of the rs1800896 polymorphism (–1082 G > A) of the *IL10* gene were not associated with the occurrence of OT. Our results contrast with the results reported by other studies. In a population from southeastern Brazil, evidence of association was found between the A allele (rs1800896) and the presence of OT (11). In Colombian patients the G/G genotype (rs1800896) and the *IL10* gene “AG” haplotype (rs1800896, rs1800871) were associated with the presence of OT (17). The A allele at position –1082 is associated with a low level of production of IL-10 (40). The discrepancies observed between studies may be caused, in part, by differences in methodology, sample size, and patient selection. In addition, additional factors that are difficult to control in the context of toxoplasmosis, such as duration of infection, may also have an effect on eye disease and serve as a bias. Moreover, as allelic frequency of the cytokine genes can differ between populations according to regional variations (41, 42), associations involving *IL1β* and *IL10* polymorphisms could result in different clinical and immune phenotypes in patients with OT from different populations.

Previous studies analyzed the influence of other cytokine polymorphisms on OT.

The C allele of the *IL-6* −174 G > C polymorphism, which is related to a decreased production of IL-6, may be associated with the occurrence of OT (18). In another study, the recurrence of OT was associated with the *IL1A* −889 C > T polymorphism (T allele), which is related to an increase in IL-1α expression (13). The A/A genotype of the interferon gamma (*IFN-γ*) gene polymorphism (position +874 T > A), which is related to reduced IFN-γ production, was associated with the presence of OT (14). A study on the tumor necrosis factor alpha (*TNF-α*) gene polymorphism (−308 G > A) did not find an association with the presence or recurrence of OT (12).

The data from this study also indicated that the homozygous T/T genotype of the *IL1β* –511 polymorphism and the heterozygous G/A genotype at position –1082 of the *IL10* gene were more frequent in individuals without the *T. gondii* infection. These links between infection and genotypes, which are associated with low or intermediate production of IL-1β and IL-10, respectively, provide evidence that variations in the genetic control of cytokine expression levels may influence the immune response in toxoplasmosis, as these levels influence systemic immune modulation, altering the number and responsiveness of host T cells. T-cell-mediated immune responses exercise a relevant influence on the suppression of *T. gondii* replication, resulting in chronic infection (4). Because of the difference in mean age, it can be presumed that control individuals were not exposed to infection by *T. gondii* and this could be a bias. However, G3 was useful for comparing the frequencies of the alleles and genes with the infected groups, as it was composed of healthy individuals.

The pathogenic mechanisms involved in the ocular form of toxoplasmosis can be explained, at least in part, by a cytokine-guided immune response (10, 21, 29). It is known that the production of certain cytokines is under genetic control. Therefore, polymorphisms in the promoter regions of cytokine genes could affect gene transcription, and, consequently, the expression of these molecules, in turn causing interindividual variations. Although IL-1β is one of the important cytokines involved in the pathogenesis of OT, the mechanism appears to be complex, involving many other cytokines and factors. Studies that aim to clarify the importance of cytokines in OT can offer perspectives for immunomodulatory strategies for the treatment of this disease. The main limitation of this study was that the expression levels of IL-1β and IL-10 were not evaluated.

In conclusion, the –511 C > T polymorphism of the *IL1β* gene seems to play an important role in the pathogenesis of OT in Brazilian individuals, whereas the –1082 polymorphism of the *IL10* gene does not seem to influence the occurrence of OT in this population.

## Data availability statement

The original contributions presented in the study are included in the article/**Supplementary Material**. Further inquiries can be directed to the corresponding author.

## Ethics statement

The studies involving human participants were reviewed and approved by the Research Ethics Committee of the Medicine School in São José do Rio Preto (CAAE 32259714.8.00005415). The patients/participants provided their written informed consent to participate in this study.

## Author contributions

CB was head of the FAMERP Toxoplasma Research Group. CB and CA contributed equally. CB and CA conceived and designed the study. FF, MP, RS, and GA selected the patients and developed the clinical diagnosis. WA and GM developed the molecular analysis. CB, CA, WA, VLP-C, and LM were responsible for the data analysis. CA, CB, and WA were responsible for writing the manuscript. All the authors approved the final version of the manuscript. All authors contributed to the article and approved the submitted version.
