# Supplementary material for: Role of interleukin 1β and interleukin 10 variants on ocular toxoplasmosis in Brazilian individuals
Source: Front Ophthalmol (Lausanne). 2023 Jun 29;3:1183167. doi: 10.3389/fopht.2023.1183167 (PMC11182258; doi:10.3389/fopht.2023.1183167)
Supplement: Supplementary file 1 [file DataSheet_1.pdf]

Supplementary table 1. Genotype and allele distribution for *IL1β* -511 and IL10 -1082 polymorphisms in individuals infected by *T. gondii* and in individuals without the infection.

GENOTYPES/ALLELES	G1 + G2	G3	p-value	OR (IC 95%)
	N = 214	N = 108		
	IL1β_rs16944 (− 511 C>T)			
	**n (%)**	**n (%)**		
**C/C**	20 (9.3)	15 (13.9)	NS	
**C/T**	171 (79.9)	69 (63.9)	0.002	2.24 (1.33-3.76)
**T/T**	23 (10.7)	24 (22.2)	0.007	0.04 (0.22-0.78)
**C**	211 (49.3)	99 (45.9)	NS	
**T**	217 (50.7)	117 (54.1)	NS	
	IL10_rs1800896 (-1082 G>A)			
**G/G**	10 (4.6)	1 (0.9)	NS	
**G/A**	198 (92.5)	106 (98.2)	0.04	0.23 (0.05-1.03)
**A/A**	6 (2.9)	1 (0.9)	NS	
**G**	112 (50.9)	108 (50.0)	NS	
**A**	108 (49.1)	108 (50.0)	NS	

G1: individuals with OT; G2: individuals without OT; G3: control group. NS: non-significant. Frequencies of genotypes and alleles were compared using chi square test for 2 x 2 contingency tables.
